# Once-weekly versus twice-weekly bortezomib in newly diagnosed multiple myeloma: a real-world analysis

**DOI:** 10.1038/s41408-024-01034-6

**Published:** 2024-03-22

**Authors:** Fieke W. Hoff, Rahul Banerjee, Adeel M. Khan, Georgia McCaughan, Bo Wang, Xiaoliang Wang, James Roose, Larry D. Anderson, Andrew J. Cowan, S. Vincent Rajkumar, Gurbakhash Kaur

**Affiliations:** 1 Myeloma, Waldenstrom’s, and Amyloidosis Program, Harold C. Simmons Comprehensive Cancer Center, UT Southwestern Medical Center, Dallas, TX USA; 2 Clinical Research Division, Fred Hutchinson Cancer Center, Seattle, WA USA; 3 Department of Haematology, St Vincent’s Hospital, Sydney, NSW Australia; 4 Willamette Valley Cancer Institute, Eugene, OR USA; 5 Flatiron Health, Inc, New York, NY USA; 6 Division of Hematology, Mayo Clinic, Rochester, MN USA

**Keywords:** Myeloma, Epidemiology

## Abstract

Induction regimens for multiple myeloma (MM) commonly include bortezomib, which has typically been administered twice weekly despite studies demonstrating comparable efficacy and less peripheral neuropathy (PN) with once-weekly bortezomib. We aimed to analyze the real-world prevalence and efficacy of once-weekly versus twice-weekly bortezomib regimens in newly diagnosed MM. We analyzed 2497 US patients aged 18–70 years treated with commercial first-line bortezomib using nationwide Flatiron Health electronic health record-derived data, including 910 (36.4%) patients who received twice-weekly and 1522 (63.2%) who received once-weekly bortezomib. Once-weekly bortezomib use increased over time, from 57.7% in 2017 to 73.1% in 2022. Multivariate analysis identified worsened performance status and more recent year of diagnosis with higher odds of receiving once-weekly bortezomib. Real-world progression-free survival (median 37.2 months with once-weekly versus 39.6 months with twice-weekly, *p* = 0.906) and overall survival (medians not reached in either cohort, *p* = 0.800) were comparable. PN rates were higher in patients receiving twice-weekly bortezomib (34.7% versus 18.5%, *p* < 0.001). In conclusion, once-weekly bortezomib is clearly associated with similar efficacy and fewer toxicities compared to twice-weekly bortezomib. Our findings support once-weekly bortezomib as a standard-of-care regimen for newly diagnosed patients with MM.

## Introduction

Multiple myeloma (MM) is a hematologic malignancy of post-germinal plasma cells [1, 2]. Outcomes in MM have drastically improved over the past two decades with the incorporation of new classes and combinations of drugs [3, 4]. Induction regimens for MM commonly include the selective 26S proteasome inhibitor bortezomib, which has typically been administered twice per week in 21-day or 28-day cycles. However, bortezomib-related peripheral neuropathy (PN) can impact quality of life and may occur more commonly in Black patients and other vulnerable populations [3, 5, 6]. Based on early studies suggesting comparable outcomes with less frequent dosing of bortezomib [7, 8], later retrospective single-center studies as well as a large systematic review have shown that once-weekly bortezomib has comparable efficacy with less PN as compared to twice-weekly bortezomib [9–11]. These findings have also been confirmed in several secondary analyses of prospective phase 3 data evaluating bortezomib-based regimens in transplant-ineligible MM patients [8, 12–14]. In addition, pharmacokinetic prediction models confirm that once-weekly bortezomib dosing constitutes an optimal therapeutic regimen with comparable antineoplastic activity but significantly reduced risk of thrombocytopenia [15].

Outside of clinical scenarios requiring swift disease control, once-weekly bortezomib has been widely adopted in real-world clinical practice. Nonetheless, most clinical trial protocols continue to dose bortezomib on a twice-weekly schedule. Previous analyses evaluating once-weekly bortezomib prescribing patterns are limited by their single-center design and use of historical data largely preceding 2018 [9, 10]. Consequently, our goal was to assess the prevalence, effectiveness, and toxicities of once-weekly versus twice-weekly bortezomib dosing regimens in a broader and more contemporary cohort of patients newly diagnosed with multiple myeloma.

## Methods

### Data source

This retrospective observational study used data from the US nationwide Flatiron Health electronic health record (EHR)-derived de-identified longitudinal database. The Flatiron Health database contains structured and unstructured data curated via technology-enabled abstraction from approximately 280 cancer clinics and 800 unique sites of care [16, 17]. Patients were included in our analysis if they had newly diagnosed MM (ICD-9 203.0x or ICD-10 C90.0x, confirmed with clinical review), were aged 18–70 years at diagnosis, had at least two EHR-documented clinic visits, and at least six months of follow-up before June 30, 2023 (data cut-off). Although the Flatiron Health database includes patients diagnosed since 2011, we only analyzed patients diagnosed on or after January 1, 2017, to focus on a more modern cohort. Given the prevalence of “VRd-lite” regimens (i.e., modified dosing of lenalidomide, bortezomib and dexamethasone) employing once-weekly bortezomib for older or frailer patients, we excluded patients a priori who were aged >70 years at diagnosis [18, 19]. All patients had documented treatment with bortezomib during their first line (1L) of treatment. Patients who received bortezomib as part of a clinical trial were excluded. This retrospective study was reviewed and exempted by the University of Texas Southwestern Institutional Review Board.

### Exposure and covariate definitions

Twice-weekly bortezomib was defined as patients for whom the most frequent interval between two doses of 1L bortezomib within a cycle was 3.0-4.9 days. Once-weekly bortezomib was defined as a corresponding interval of 5.0–9.9 days. The frequency of patients who started with one prescribing pattern and switched to the other pattern was noted; however, these patients were excluded from subsequent analyses. Key covariates included age at diagnosis, gender, race/ethnicity (non-Latinx White, non-Latinx Black, Hispanic/Latinx, non-Latinx Asian, other and unknown), practice type (academic, community, both), insurance status, Eastern Cooperative Oncology Group (ECOG) performance status (PS) at 1L initiation (0, 1, ≥2, unknown), ISS stage (I, II, III, unknown/not documented) [20], year of diagnosis, serum creatinine at treatment initiation ( ≤ 1.2 mg/dL, 1.3–2.9 mg/dL, ≥3 mg/dL, and unknown), hemoglobin (g/dL) level at treatment initiation, number of high-risk cytogenetic abnormalities (HRCA) tested any time prior to or within days of 1 L initiation (HRCA 0, 1, ≥2), and route of first dose of bortezomib (subcutaneous versus intravenous). HRCA included gain(1q)/amp(1q), t(4;14), t(14;16), t(14;20), and del(17p) [21, 22]. Insurance status was categorized hierarchically as Medicaid, Medicare, Commercial Health Plan, other, or unknown/uninsured; for patients aged ≥65 years at diagnosis with missing insurance status, insurance was assumed to be Medicare.

Concurrent anti-MM treatments alongside bortezomib were defined as follows: bortezomib only, cyclophosphamide-bortezomib-dexamethasone (CyBorD), daratumumab-bortezomib-dexamethasone (D-Vd), daratumumab-bortezomib-lenalidomide-dexamethasone (D-VRd), bortezomib-dexamethasone (Vd), bortezomib-lenalidomide-dexamethasone (VRd), and other. Neuropathy during 1L treatment was defined as the presence of at least one of the following: billing codes for drug-induced neuropathy (ICD-9 357.6, ICD-10 G62.2), billing codes for other neuropathies (ICD-9: 356.9, 357.4, 357.7; ICD-10: G62.9), and initiation of medications typically reserved for neuropathy (gabapentin, pregabalin, and/or duloxetine).

### Endpoints and statistical analysis

Real-world overall survival (rwOS) was defined as the time from start of first treatment (index date) to the date of death or last confirmed activity before the data cut-off [23, 24]. Real-world progression free survival (rwPFS) was defined as time from start of first treatment to the first derived date of progressive disease, death, or last confirmed activity before the data cut-off. Disease progression status was derived using International Myeloma Working Group (IMWG) criteria [25] using the results of serum protein electrophoresis (SPEP) testing (abstracted from unstructured health record information if value ≥1.0 g/dL at baseline), 24-h urine protein electrophoresis (UPEP) testing (abstracted from unstructured health record notes if value ≥200 mg per 24 h at baseline), and serum free light chain (FLC) testing (abstracted from structured laboratory information). Patients with no documented progression or death were censored at the date of the last test of the assigned biomarker type.

Descriptive statistics were used to describe demographic, clinical, disease, and treatment characteristics. Continuous variables were reported as means, medians, and interquartile ranges (IQRs). Categorical variables were reported as number (n) and percent (%) of eligible patients. We used multivariate logistic regression to assess demographic and clinical factors associated with once-weekly versus twice-weekly (reference) bortezomib. Estimated adjusted odds ratios (OR) and corresponding 95% confidence intervals (CI) were summarized. The Kaplan-Meier method was used to estimate rwOS and rwPFS. Outcomes were compared using Cox proportional hazards (PH) models including stem cell transplant as a time-varying covariate and adjustment for other demographic and clinical factors. Adjusted hazard ratio (HR) and corresponding 95% CI of once-weekly bortezomib, as compared to twice-weekly bortezomib, were summarized for each outcome. The PH assumption of the bortezomib prescribing pattern coefficient was tested using a score test in the multivariate model with time-varying transplant variable. All statistical analyses were performed using R version 4.1.3.

## Results

**Fig. 1:**
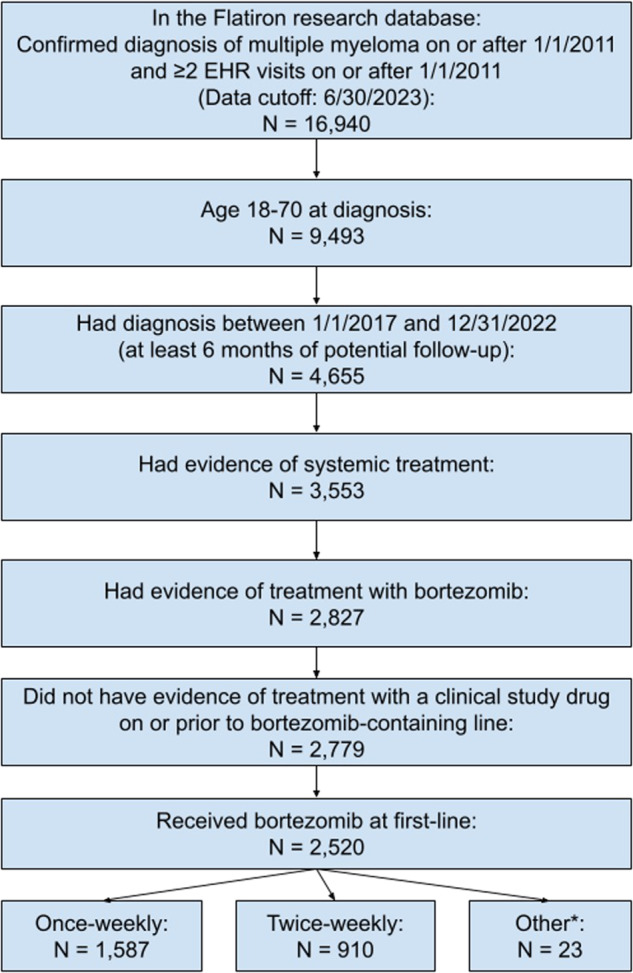
Flow chart of analyzed patients. Abbreviations: EHR, Electronic Health Record. * *Other*: Patients whose bortezomib dosing frequency changed during induction.

As shown in Fig. 1, we included 2520 patients diagnosed with MM between 2017 and 2022 who received 1L bortezomib. Twenty-three patients (0.9%) were excluded due to changing bortezomib frequencies during 1L, leaving 2497 (99.1%) evaluable patients. Of these 2497 patients, 910 (36.4%) received twice-weekly bortezomib and 1587 (63.6%) received once-weekly bortezomib. Patients had a median age at diagnosis of 62 years (IQR: 56–67 years) and 44.6% of the patients were female. Approximately half of patients (49.7%) were non-Latinx White, 22.1% were non-Latinx Black, and 8.5% were Latinx. Most patients were treated in the community setting (77.1%) (Table 1).

**Table 1:** Baseline characteristics between twice-weekly and once-weekly bortezomib.

	ALL	Twice-weekly	Once-weekly	*p*-value
	*N* = 2497	*N* = 910	*N* = 1587	
Age at index	62.0 [56.0;67.0]	62.0 [57.0;67.0]	62.0 [56.0;67.0]	0.774
Gender				0.478
Male	1383 (55.4%)	513 (56.4%)	870 (54.8%)	
Female	1114 (44.6%)	397 (43.6%)	717 (45.2%)	
Race/ethnicity				<0.001
Non-Latinx White	1242 (49.7%)	467 (51.3%)	775 (48.8%)	
Non-Latinx Black	552 (22.1%)	208 (22.9%)	344 (21.7%)	
Hispanic or Latinx	212 (8.5%)	102 (11.2%)	110 (6.9%)	
Non-Latinx Asian	58 (2.3%)	14 (1.5%)	44 (2.8%)	
Other	117 (7.1%)	52 (5.7%)	125 (7.9%)	
Unknown	256 (10.3%)	67 (7.4%)	189 (11.9%)	
Practice type				0.040
Community	1925 (77.1%)	723 (79.5%)	1202 (75.7%)	
Academic	451 (18.1%)	154 (16.9%)	297 (18.7%)	
Both	121 (4.8%)	33 (3.6%)	88 (5.5%)	
Year of diagnosis	2019 [2018;2021]	2019 [2018;2021]	2020 [2018;2021]	<0.001
ECOG PS at 1L				0.016
0	839 (33.6%)	334 (36.7%)	505 (31.8%)	
1	782 (31.3%)	288 (31.6%)	494 (31.1%)	
≥2	344 (13.8%)	105 (11.5%)	239 (15.1%)	
Unknown	532 (21.3%)	183 (20.1%)	349 (22.0%)	
ISS stage				0.180
Stage I	685 (27.4%)	265 (29.1%)	420 (26.5%)	
Stage II	516 (20.7%)	181 (19.9%)	335 (21.1%)	
Stage II	533 (21.3%)	205 (22.5%)	328 (20.7%)	
Unknown	763 (30.6%)	259 (28.5%)	504 (31.8%)	
Insurance status at 1L				0.905
Commercial Health Plan	778 (31.2%)	276 (30.3%)	502 (31.6%)	
Medicare	798 (32.0%)	300 (33.0%)	498 (31.4%0	
Medicaid	191 (7.6%)	67 (7.4%)	124 (7.8%)	
Other payer	219 (8.8%)	82 (9.0%)	137 (8.6%)	
Unknown/ uninsured	511 (20.5%)	185 (20.3%)	326 (20.5%)	
Number of HRCA				0.266
0	1733 (69.4%)	619 (68.0%)	1114 (70.2%)	
1	564 (22.6%)	208 (22.9%)	356 (22.4%)	
≥2	200 (8.0%)	83 (9.1%)	117 (7.4%)	
Serum creatinine level at 1L				0.802
≤1.2 mg/dL	1216 (48.7%)	434 (47.7%)	782 (49.3%)	
1.3–2.9 mg/dL	494 (19.8%)	183 (20.1%)	311 (19.6%)	
≥3 mg/dL	202 (8.1%)	79 (8.7%)	123 (7.8%)	
Unknown	585 (23.4%)	214 (23.5%)	371 (23.4%)	
Hemoglobin level (g/dL)	10.7 [9.1;12.3]	10.6 [9.0;12.3]	10.7 [9.2; 12.3]	0.385
Concurrent therapy at 1L				
Bortezomib only	27 (1.1%)	9 (1.0%)	18 (1.1%)	
CyBorD	305 (12.2%)	74 (8.1%)	231 (14.6%)	
D-Vd	41 (1.6%)	8 (0.9%)	33 (2.1%)	
D-VRd	276 (11.1%)	83 (9.1%)	193 (12.2%)	
Other	5 (0.2%)	< 5	< 5	
Vd	130 (5.2%)	54 (5.9%)	76 (4.8%)	
VRd	1713 (68.6%)	681 (74.8%)	1032 (65.0%)	
Bortezomib starting routes at 1L				<0.001
Subcutaneous	2368 (94.9%)	841 (92.4%)	1527 (96.3%)	
Intravenous	128 (5.1%)	69 (7.6%)	59 (3.7%)	
Any neuropathy diagnosis or medication for severe neuropathy	610 (24.4%)	316 (34.7%)	294 (18.5%)	<0.001
Drug-induced neuropathy diagnosis	134 (5.4%)	87 (9.6%)	47 (3.0%)	<0.001
Other neuropathy diagnosis	141 (5.6%)	78 (8.6%)	63 (4.0%)	<0.001
Medication for severe neuropathy	506 (20.3%)	272 (29.9%)	234 (14.7%)	<0.001

*ECOG* Eastern Cooperative Oncology Group, *PS* performance status, *HRCA* high-risk cytogenetic abnormality, *CyBorD* cyclophosphamide-bortezomib-dexamethasone, *D-Vd* daratumumab-bortezomib-dexamethasone, *D-VRd* daratumumab-bortezomib-lenalidomide-dexamethasone, *Vd*: bortezomib-dexamethasone, *VRd* bortezomib-lenalidomide-dexamethasone.

**Fig. 2:**
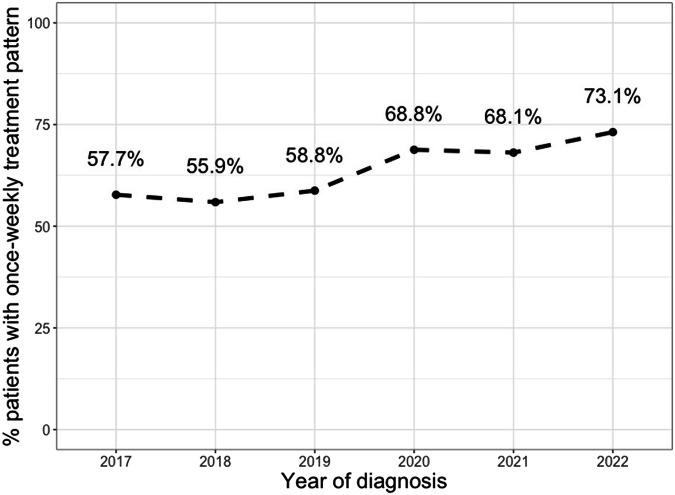
Proportion of patients treated with once-weekly bortezomib.

Compared to patients with twice-weekly bortezomib, patients who received once-weekly bortezomib were less likely to be Hispanic/Latinx (6.9% versus 11.2%, *p* < 0.001). Conversely, they were more likely to be treated at academic practices (18.7% versus 16.9%, *p* = 0.040), have ECOG PS ≥ 2 (15.1% versus 11.5%, *p* = 0.016), and to have started bortezomib subcutaneously (96.3% versus 92.4%, *p* < 0.001) (Table 1). Other demographic and clinical characteristics were similar between the two groups, including age, gender, ISS stage, hemoglobin, number of HRCAs and concurrent therapies. The frequency of once-weekly bortezomib use did increase over time, from 57.7% in 2017 to 73.1% in 2022. In addition, we observed a step up in once-weekly dosing frequency between 2017–2019 (57.5% of eligible patients) versus 2020-2022 (69.9% of eligible patients (Fig. 2).

In multivariate logistic regression analysis, ECOG PS ≥ 2 (OR 1.57 [95% CI 1.19-2.08] versus ECOG PS 0), and more recent year of diagnosis (OR 1.18 [95% CI 1.12–1.24] per year) were associated with higher odds of receiving once-weekly bortezomib (Table 2). A trend was found between twice-weekly bortezomib and ≥2 HRCA at 1L (OR 0.74 [0.54-1.00] versus 0 HRCA). Conversely, once-weekly bortezomib administration was less common among Hispanic/Latinx patients compared to non-Latinx White (OR 0.65 [95% CI 0.48–0.89]) and among patients dosed intravenously rather than subcutaneously (OR 0.44 [95% CI 0.30–0.65]) had lower odds of receiving once-weekly bortezomib administration. No significant associations were found between gender, ISS stage, practice setting, insurance category, or baseline creatinine.

**Table 2:** Predictors of once-weekly bortezomib dosing.

Characteristic	OR^a^	95% CI^a^	*p*-value
Age at diagnosis	1.00	0.99, 1.01	0.817
Gender			
Male	–	–	
Female	1.07	1.27	0.443
Race/ ethnicity			
Non-Latinx White	–	–	
Non-Latinx Black	0.95	0.76, 1.17	0.607
Hispanic or Latinx	0.65	0.48, 0.89	0.007
Non-Latinx Asian	1.98	1.08, 3.84	0.033
Other	1.55	1.10, 2.23	0.015
Unknown	1.69	1.24, 2.33	<0.001
Practice type			
Community	–	–	
Academic	1.14	0.90, 1.43	0.275
Both	1.76	1.17, 2.72	0.009
ECOG PS			
0	–	–	
1	1.16	0.94, 1.43	0.165
≥2	1.57	1.19, 2.08	0.002
Unknown	1.29	1.02, 1.66	0.037
ISS stage			
Stage I	–	–	
Stage II	1.17	0.92, 1.50	0.205
Stage II	1.03	0.79, 1.34	0.855
Unknown	1.29	1.02, 1.62	0.031
Insurance at 1L			
Commercial Health plan	–	–	
Medicare	0.97	0.76, 1.24	0.809
Medicaid	0.98	0.69, 1.39	0.908
Other payer	1.01	0.74, 1.40	0.935
Unknown/ uninsured	1.09	0.86, 1.39	0.475
Number of HRCA at 1L			
0	–	–	
1	0.95	0.77, 1.16	0.598
≥2	0.74	0.54, 1.00	0.051
Diagnosis year	1.18	1.12, 1.24	<0.001
Serum creatinine level at 1L			
≤1.2 mg/dL	–	–	
1.3–2.9 mg/dL	0.95	0.75, 1.21	0.689
≥3 mg/dL	0.83	0.59, 1.17	0.277
Unknown	1.02	0.82, 1.26	0.877
Bortezomib starting routes			
Subcutaneous	–	–	
Intravenous	0.44	0.30, 0.65	<0.001

*OR* odds ratio, *CI* confidence interval, *ECOG* Eastern Cooperative Oncology Group, *PS* performance status, *ISS* International Staging System, *HRCA* high-risk cytogenetic abnormality.

^a^Adjusted for age at diagnosis, gender, race/ethnicity, practice type, ECOG PS, ISS stage, insurance, number of HRCAs, year of diagnosis, creatinine level and treatment starting route; Treatment reference group: Twice-weekly prescribing at 1L.

**Fig. 3:**
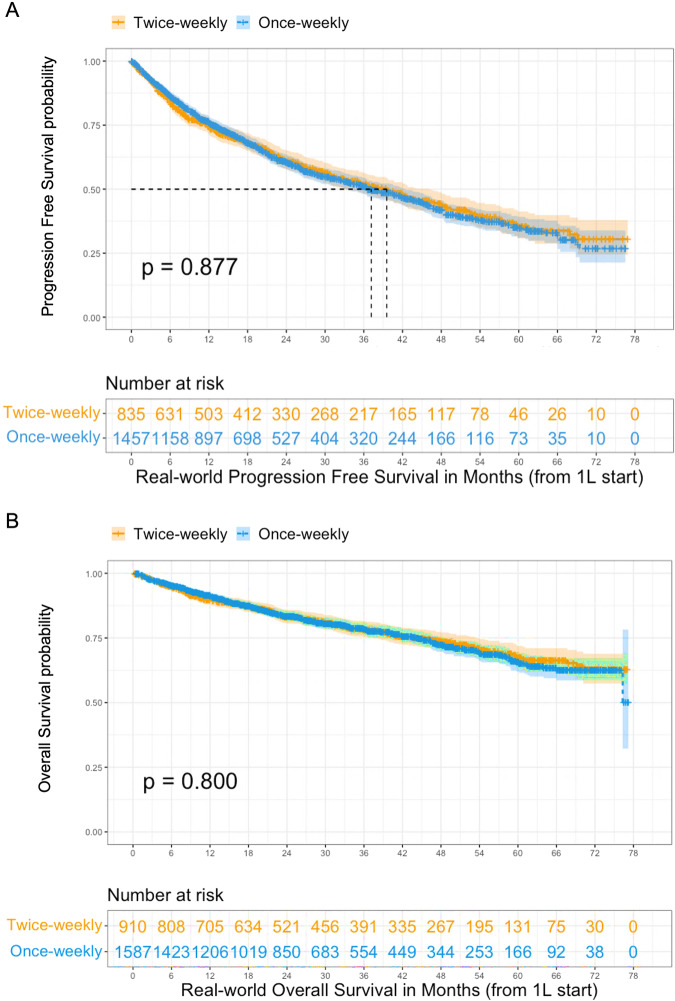
Real-world PFS and OS based on bortezomib dosing frequency. **A** Real-world PFS. **B** Real-world OS.

As shown in Fig. 3A, there was no statistically significant difference in rwPFS among patients receiving once-weekly (median 37.2 months, 95% CI 33.1–42.4 months) versus twice-weekly bortezomib (median 39.6 months, 95% CI 33.2-46.1 months). The adjusted HR was 0.90 with 95% CI 0.79–1.03 (Table 3). As shown in Fig. 3B, there was also no statistically significant difference in rwOS with median 27.1 months follow-up. Median rwOS was not reached in either group, with a HR 0.90 [95% CI 0.75–1.08]. No violations of the PH assumption were observed. The cumulative neuropathy rate, encompassing any PN diagnosis or initiation of medications for neuropathy was 24.4% overall. As shown in Table 1, PN was significantly more common with twice-weekly bortezomib than once-weekly bortezomib (34.7% versus 18.5%, *p* < 0.001).

**Table 3:** Associations between bortezomib dosing and survival.

	Number of patients	Median (months) (95% CI)	HR^a^(95% CI)	*p*-value
rwPFS				
Twice-weekly	835	39.6 (33.2–46.1)	1.00 (ref)	0.135
Once-weekly	1457	37.2 (31.1–42.4)	0.90 (0.79–1.03)	
rwOS				
Twice-weekly	910	NA (NA–NA)	1.00 (ref)	0.271
Once-weekly	1587	NA (76.3–NA)	0.90 (0.75–1.08)	

^a^Adjusted for age at diagnosis, gender, race/ethnicity, practice type, ECOG PS, ISS stage, insurance, number of HRCAs, year of diagnosis, creatinine level and treatment starting route; Treatment reference group: Twice-weekly prescribing at 1L.

## Discussion

The incorporation of bortezomib into MM treatment regimens represents a major advancement in the management of this disease. In the frontline setting, for example, the SWOG S0777 and IFM 2005-01 trials demonstrated an overall survival benefit with the addition of twice-weekly bortezomib to induction regimens [26, 27]. Compared to its initial administration in SWOG S0777 and other early trials, bortezomib has largely shifted from intravenous to subcutaneous dosing based on the results of non-randomized analyses showing similar efficacy with fewer toxicities [28]. Indeed, over 90% of patients in our real-world analysis received subcutaneous bortezomib. However, clinical trials have largely continued to use twice-weekly bortezomib dosing even with the transition to subcutaneous dosing. Apart from the ALCYONE and BOSTON studies, relatively few Phase 3 trials in the past decade have incorporated once-weekly bortezomib [29, 30]. Several small studies from academic centers and a large systematic review have already shown that once-weekly dosing has similar efficacy [9–11]. We aimed to study this question with a much larger data set including patients treated in the community setting, who in fact comprised over 75% of our analyzed population.

The frequency of once-weekly bortezomib gradually increased over time, with the majority (73.1%) of patients receiving once-weekly bortezomib in 2022. The usage of once-weekly bortezomib in our data set is higher than these reported in two previous retrospective analyses evaluating bortezomib administration between 2005-2013 and 2008-2018, both of which showed that twice-weekly bortezomib was being prescribed roughly as often as once-weekly bortezomib regimens [9, 10]. This trend may reflect a growing consensus in the field that once-weekly bortezomib constitutes a standard-of-care regimen for MM [31]. Alternatively, some of these differences are likely attributable to the COVID-19 pandemic and a desire to minimize infectious exposures during each clinic visit for bortezomib injection. Indeed, a recent study examining over 7000 patients with MM using similar Flatiron Health data showed significant reductions in all types of cancer-related visits beginning in March 2020 with the pandemic [31, 32].

That being said, approximately a quarter of patients in our study continued to receive bortezomib dosed twice-weekly. Patients who received twice-weekly bortezomib were more likely to be Hispanic/Latinx and less likely to be Asian, a finding broadly in line with previous analyses of racial and ethnic disparities in bortezomib dosing [33]. Additionally, the use of twice-weekly bortezomib was less likely in patients with worsened performance status. While practice setting did not remain associated in multivariate logistic regression analysis, patients treated in community settings were slightly more likely to receive twice-weekly bortezomib. Twice-weekly bortezomib is often utilized in the setting of acute cast nephropathy, which necessitates rapid disease control; however, no relationship was found between creatinine levels at diagnosis and bortezomib dosing. While HRCAs were significantly more common among patients receiving twice-weekly bortezomib, this was not statistically significant in the multivariate analysis.

Regarding clinical outcomes, once-weekly and twice-weekly bortezomib demonstrated comparable rwPFS and rwOS: The median rwPFS was 38.1 months versus 39.8 months, and the median rwOS was not reached in either treatment group. This observation is consistent with outcomes/results of other smaller, single-center studies [9, 10, 12]. Fewer than 1% of patients in our analysis switched from twice-weekly to once-weekly bortezomib during the course of 1 L treatment, making this unlikely as a factor that might have affected the overlapping efficacy of different bortezomib dosing schedules. With regard to neuropathy defined either by billing codes or by the initiation of medications such as gabapentin, we found PN incidences of 18.5% with once-weekly bortezomib versus 34.7% with twice-weekly bortezomib. This matches the conclusion of a previous analysis of pooled data from Phase 3 trials; however, that study used physician determinations of PN and showed a 32% incidence with once-weekly versus 47% with twice-weekly bortezomib [12].

While this represents the largest and the most extensive real-world analysis of bortezomib dosing to date, our retrospective study is not without its limitations. Although we used direct laboratory values and abstracted chart data to assess IMWG responses rather than relying on physician documentation, we did not have full access to full imaging reports to corroborate responses in patients with extramedullary disease. Due to the nature of the Flatiron Health database, PN was identified based on surrogate measures of a combination of diagnostic codes for neuropathy and PN-associated medications. It is probable that patients with mild PN may have gone unreported or undocumented. Indeed, the frequency of initiation of PN-associated medications was approximately four times as high as that of documented PN diagnoses. Furthermore, there may have been unmeasured confounders that influenced physician decision-making around bortezomib dosing. A randomized controlled trial directly comparing once-weekly bortezomib versus twice-weekly bortezomib would be ideal approach to address this question. However, given the overwhelming preference for once-weekly bortezomib dosing among 90% of physicians across the world in a recently published international survey [31], there would not be equipoise for such a study to be launched today.

In conclusion, this study supports the incorporation of once-weekly bortezomib into standard-of-care regimens for newly diagnosed patients with MM. Just as with non-randomized comparisons of subcutaneous versus intravenous bortezomib, once-weekly bortezomib is associated with equivalent outcomes and a more favorable side-effect profile compared to twice-weekly dosing. More broadly, once-weekly bortezomib not only reduces the clinical burden of care by reducing visit frequency but may likely be more cost-effective as well.

## Data Availability

The data that support the findings of this study have been originated by Flatiron Health, Inc. Requests for data sharing by license or by permission for the specific purpose of replicating results in this manuscript can be submitted to dataaccess@flatiron.com.
